# Comparing the Outcomes of Early and Late Tracheostomy in Severe Traumatic Brain Injury Patient

**DOI:** 10.21315/mjms2021.28.4.7

**Published:** 2021-08-26

**Authors:** Muhammad Ihfaz Ismail, Zamzuri Idris, Jafri Malin Abdullah, Noor Azman A Rahman, Mazin Nordin

**Affiliations:** 1 Department of Neurosciences, School of Medical Sciences, Universiti Sains Malaysia, Kubang Kerian, Kelantan, Malaysia; 2 Department of Neurosurgery, Hospital Sultanah Aminah, Johor Bahru, Johor, Malaysia; 3 Department of Radiology, Hospital Sultanah Aminah, Johor Bahru, Johor, Malaysia; 4 Hospital Universiti Sains Malaysia, Universiti Sains Malaysia, Kubang Kerian, Kelantan, Malaysia; 5 Brain Behaviour Cluster, School of Medical Sciences, Universiti Sains Malaysia, Kubang Kerian, Kelantan, Malaysia

**Keywords:** severe traumatic brain injury, Neurosurgical High Dependency Unit, Glasgow Coma Scale, tracheostomy, ventilator associated pneumonia

## Abstract

**Background:**

Patients with severe traumatic brain injury (TBI) were expected to have poor Glasgow Coma Scale (GCS) recovery and prolonged intubation. Therefore, an early tracheostomy procedure was indicated for all severe TBI. In view of growing concern regarding the safety and outcome of early tracheostomy on these patients, it was deemed valid and needed to be addressed.

**Methods:**

This study was conducted to compare the outcomes of early and late tracheostomies in severe TBI. Only severe TBI patients who were admitted to the Neurosurgery High Dependency Unit (NHDU), Hospital Sultanah Aminah (HSA), Johor Bahru, Johor, Malaysia and who had underwent a tracheostomy were recruited. Three main outcomes noted: duration on ventilation, length of NHDU stay and rate of ventilator associated pneumonia (VAP).

**Results:**

Out of 155 patients, 72 (46.5%) were in early tracheostomy group (ETG) and 83 (53.5%) were in late tracheostomy group (LTG). The majority of the participants, 95 (61.3%) were ethnic Malays. The mean duration on ventilator use was 2.65 days (1.57) for ETG and 5.63 days (2.35) for LTG. While, mean NHDU stay was 4.75 days (1.98) for ETG and 9.77 days (2.70) for LTG. Upon independent *t*-test, early duration of tracheostomies had shown significant outcome in reducing length of NHDU stay, (*P* < 0.001) and had shortening participants’ time on mechanical ventilator (*P* < 0.001). Then, based on forward multiple logistic regression test, there were significant association between comorbid (*P* = 0.003) and tracheostomy (*P* = 0.020) towards presence of VAP when adjusted for other variables.

**Conclusion:**

In this study it was found that early tracheostomy was significant in shortening the duration on ventilator, reducing the length of NHDU stay and reducing the rate of VAP.

## Introduction

Tracheostomy is a simple procedure conducted for the purpose of securing the airway, performed usually on severe traumatic brain injury (TBI) patient. A tracheostomy has many advantages over translaryngeal endotracheal intubation including reduced laryngeal ulceration, reduce respiratory resistance and making nursing care easier while facilitating airway suctioning, mouth care and patient mobility (1, 2). Head injury is defined as blunt and/or penetrating injury to the head and/or brain due to an external force with temporary or permanent impairments to the brain function which may or may not result in underlying structural changes in the brain (3). Head injury are classified into mild, moderate and severe according to Glasgow Coma Scale (GCS) score. A patient presenting with a severe head injury is expected to have poor GCS recovery and prolonged intubation; therefore, a tracheostomy is a must for all severe TBI patients. The Hospital Sultanah Aminah (HSA), Johor Bahru, Johor is located at the southern part of Malaysia and is a tertiary referral centre for the neurosurgical cases. It is equipped with Neurosurgery High Dependency Unit (NHDU) ward with 10 beds and 8 mechanical ventilators available. There is high turnover of patients needing neurosurgical care since this NHDU ward caters emergency as well as elective cases, including brain and spine cases. The optimal timing of an early tracheostomy is still a controversial issue. For example, there is no uniformity in the literature about the definition of ‘early’ tracheostomy. In the 1980s, a tracheostomy was considered ‘early’ if it was performed before 21 days of translaryngeal intubation (2). Over the last several years, however, the timing of tracheostomy has changed and now many suggest tracheostomy within 2–10 days (2).

This definition of ‘early’ corresponds to that proposed by the Otorhinolarygologists, who have always suggested tracheostomy within several days to prevent laryngeal injury from even these short periods of intubation (4). According to Durbin et al. (4), early tracheostomy defined as within 3–5 days of translaryngeal intubation. In this present study, early tracheostomy is defined as a tracheostomy procedure performed within three days after patients undergoes translaryngeal intubation.

The aim of this study was to compare the outcomes of early and late tracheostomies concerning duration of mechanical ventilator use, length of stay in NHDU from date of tracheostomy was performed and the rate of ventilator associated pneumonia (VAP). In view of growing concern regarding the safety and outcomes of early tracheostomy on these patients, it was deemed valid and it needed to be addressed.

## Methods

This study was a retrospective cohort on patients with severe TBI which was conducted for 20 months from January 2016 to September 2017. The study proposal was beforehand sent for approval from the Malaysia Medical Research and Ethics Committee (MREC).

### Study Participants

In this study, it started with selecting all the TBI patient who underwent tracheostomy who fulfilled the inclusion and exclusion criteria. From this, patient further divided retrospectively into two groups; early tracheostomy group (ETG) and late tracheostomy group (LTG). Early tracheostomy was defined as tracheostomy performed within first three days after translaryngeal intubation, while late tracheostomy defined as tracheostomy performed after day 4 of translaryngeal intubation.

The inclusion criteria for this study were: i) patients with severe TBI with an abbreviated injury scale (AIS) ≥ 4; ii) patients who had underwent tracheostomy procedure during their stay in the NHDU; iii) only patients admitted to NHDU; iv) patients with a clear history of trauma and v) patients between the age > 18 years old and < 65 years old.

The exclusion criteria were: i) patients with severe chest injury with an AIS score ≥ 4 (e.g. flail chest) and ii) patients with severe cervical injury with an AIS ≥ 4 (e.g. unstable cervical fracture). Patients presenting these two conditions were expected to have ventilation issue which would prolonged duration on mechanical ventilation. Participants were identified from the operation theatre book registry and NHDU admission database and those who fulfilled both the inclusion and exclusion criteria were included in this study.

The unique features differentiating this study which differ from others study on the outcomes of tracheostomies in patients with severe TBIs are that the study was conducted only in NHDU which is mainly managed by neurosurgeons and that the setting of mechanical ventilators was co-managed with an anaesthetic team. Furthermore, the decision to proceed with tracheostomy and when to wean patient off the ventilator after tracheostomy were decided by neurosurgeons. Most of the tracheostomies were performed by a neurosurgical trainee either by medical officer except for cases which required more expertise and experience that were performed by an otorhinolaryngology team. The patients’ baseline demographic and pre-existing condition were recorded. Clinical status at the presentation in the form of GCS, pupil response to light and injury severity score (ISS) were documented.

The computed tomography (CT) brain scans of the participants involved in this study were classified according to Marshall classification and along with surgeon who performed the tracheostomy, complications of tracheostomy, smoking habits and clinical status upon discharge from NHDU was collected and documented to describe the variability of study population.

The ISS is an anatomical scoring system that provide an overall score for patient with multiple injuries. In this study each injury was assigned an AIS score and was allocated to one of six body regions (head, face, chest, abdomen, extremities [including pelvis] and external). Only the highest AIS score in each body region was used. The three most severely injured body regions have their score squared and added together to produce the ISS score. Nowadays it is very rare to have an isolated severe head injury in a motor-vehicle accident.

The ventilator associated pneumonia (VAP) is defined as a pneumonia acquired when a patient is on mechanical ventilation for more than 48 h on the date of event, with the day of ventilator placement being day 1 (5). The criteria for a VAP diagnosis are: i) radiographic evidence for two or more serial chest radiograph with at least one of the following: a) new or progressive and persistent infiltrate, b) consolidation and c) cavitation. In a patient without underlying pulmonary or cardiac disease (e.g. respiratory distress syndrome, bronchopulmonary dysplasia, pulmonary oedema or chronic obstructive pulmonary disease), one definitive chest radiograph is acceptable; ii) clinical signs that include at least one of the following: a) fever (38 °C) with no other recognised cause, b) leucopenia (< 4000 white blood count [WBC]) or leukocytosis (> 12000 WBC), c) for adult > 70 years old, altered mental status with no other recognised cause and iii) at least two of the following: a) new onset of purulent sputum or change in character of sputum, b) new onset or worsening of cough or dyspnea or tachypnea, c) rales or bronchial breath sounds, d) worsening of gas exchange (e.g. O_2_ desaturation [e.g. PaO_2_:FiO_2_ ratio < 240]) and e) increased oxygen requirement or increased ventilator demand (5).

The chest radiograph were collected as one of the criteria to diagnose VAP and were interpreted by a radiologist blinded to without whether patients were in the ETG or LTG.

### Sample Size

The duration of data collection was from 1 January 2016 to 30 September 2017. All participants who met both inclusion and exclusion criteria during that period were recruited. The standard deviation (SD) of intensive care unit (ICU) stay and duration of use of mechanical ventilator use were based on clinical article by Shamim et al. (6). It was estimated that from a sample size of about 110, including 10% dropout, had derived a value of SD of 3.21 days for ICU stay, a difference of two days between early and late tracheostomies would be derived. This resulted in a significance level of 0.05 and power of study of 80%. For the duration of time on mechanical ventilator, an estimated sample size of about 60, including 10% dropout, produced a SD of 2.4 days. This gave a difference of two days between early and late tracheostomies, with a significance level of 0.05 and power of study of 80%. The rate of pneumonia is based on Rodriguez et al. (7). The estimated sample size including 10% dropout was 150, while the proportion pneumonia in patients undergoing early tracheostomies, P_0_ was 0.76, while the proportion of pneumonia in patients undergoing late tracheostomies, P_1_ was 0.98 with a significance level of 0.05 and power of study of 80%. All these calculations had utilised a software of power and sample size programme version 3.05, while the sample size was estimated in accordance to the rate of pneumonia since it was the largest sample size. Statistical analysis was performed using Statistical Package for Social Science (SPSS) version 24 software. The patient demographics are presented in table form using mean and SD for the numerical variables, as well as number and percentages for the categorical variables. An independent *t*-test was used in order to determine the outcome for length of NHDU stay and duration of ventilator use, while a forward multiple logistic regression test was used to determine which other factors can influence the rate of VAP. A statistical significance was considered when *P* was ≤ 0.05. The patients who were recruited for this study would proceed with follow-up until they were discharged from the NHDU prior to leaving the hospital. Information on patients: duration of use on mechanical ventilator, length of NHDU stay and incidence of VAP while staying in NHDU, were recorded. The ultimate aim of this study conducted, was to compare the outcomes of ETG and LTG with severe TBI, who were treated only in the NHDU, HSA, Johor Bharu, Malaysia.

## Results

A total number (*n*) of 155 patients, were collected retrospectively from January 2016 until September 2017. The data were traced manually from operation theatre registry and NHDU admission database. Table 1 summarise the demographic of population studied. Out of 155 (*n*) patients, 135 (87.1%) were male and 20 (12.9%) were female. The population ranged from 18 to 65 years old with the mean of 37.5 (SD = 16.76). Malays comprised majority of the patients, with 95 (61.3%), followed by Chinese, 38 (24.5%), Indian, 6 (3.9%) and others 16 (10.3%). There were 72 (46.5%) early tracheostomy patients and 83 (53.5%) late tracheostomy patients. About 142 (91.6%) of the patients were not associated with lung disease and only 13 (8.4%) associated with lung disease. There were non-lethal complication of tracheostomies: one (0.6%) case for infected tracheostomy wound and three (1.9%) cases of subcutaneous emphysema. Seven (4.5%) subjects died upon discharge from the NHDU but their death were not related to their tracheostomies.

The mean of NHDU stay was 7.44 days (SD = 3.46) and the mean duration on ventilation was 4.25 days (SD = 2.51). Out of 155 patients underwent a tracheostomy, 136 (87.7%) did not contract VAP and only 19 (12.3%) had their condition complicated with VAP. Pre-operative pupils were equal in 112 (72.3%), while unequal pupils were observed in 43 (27.7%) of the participants. The CT brain scans of patients with severe TBIs were classified according to Marshall classification. Marshall classification type 3 was the common finding, 72 (49.7%) among participants in this study.

As depicted on Table 2, there were significant mean difference in both days in the NHDU and duration on a ventilator in days when comparing the ETG and LTG (*P* < 0.001). Patients in LTG spent on average 5.02 more days in the NHDU (mean difference [95% CI: −5.77, −4.28]). The mean duration of patient in the LTG for days on a ventilator was 5.63 (95% CI: 5.19, 6.07) which was higher than the ETG, 2.65 days (95% CI= −3.62, −2.33).

As can be seen in Table 3, there were significant association between comorbidity (*P* = 0.003) and tracheostomy (*P* = 0.020) concerning the presence of VAP when adjusted for other variables. Patients with lung disease, were 6.92 times more likely to present with VAP compared to patients with no lung disease (odds ratio [OR] = 6.92; 95% CI: 1.92, 24.86) while patients undergoing a late tracheostomy, were 4.71 times more likely present with VAP compared to patient who underwent an early tracheostomy (OR = 4.71; 95% CI=1.27, 17.41).

## Discussion

This study revealed the outcomes of early and late tracheostomies in patients with severe TBIs, who were admitted to the NHDU, HSA, Johor Bahru. With the vast increase in TBI cases every year, the demand for neurosurgical services had increased exponentially.

According to the Health Facts, Ministry of Health (MOH) Malaysia, TBI was documented as fifth commonest cause of hospitalisation in MOH hospital for the year of 2014 (7.86%) and 2015 (7.64%) (8, 9). Due to the limited beds and mechanical ventilators available in NHDU and great demand for neurosurgical services, there is a necessity to subject all severe TBI patient for early tracheostomy.

Severe TBI patients were expected to have poor GCS recovery and are projected for prolonged stays on mechanical ventilator. Therefore, a tracheostomy offers the potential benefits of less need for sedation and lower airway resistance (than through an endotracheal tube), that can facilitate the weaning process off of a mechanical ventilator and leading to shorter NHDU stays. In addition, it can improve patient comfort, provide the opportunity for oral feeding, enable the ability to communicate and create condition for easier well as easier and safer nursing care. Moreover, by preventing micro-aspiration of secretions, tracheostomy can reduce the incidence of VAP (4).

Head injury patients are considered a major international cause of morbidity, mortality and socioeconomic cost (10). These consequences, indicate the importance of prevention efforts in terms of increasing public awareness, road safety modifications, raising car and motorcycle safety profiles and safety awareness among construction workers.

According to You et al. (11) with each day of ICU stay, the cost of care is significantly increased (by RM2,645.42 per day) and patients who contracted pneumonia had a significantly higher mean cost than those who did not contract pneumonia (difference of RM31,100.42).

In the present study, an early tracheostomy in patients with severe TBI was shown to reduce the length of NHDU stay by an average 5.02 days and to shorten patient time on a ventilator to as little as 2.98 days. Additionally, early tracheostomies showed a strong association with reducing the rate of VAP.

Overall, the outcomes of this study can be very beneficial to overcoming the shortage of neurosurgical care in the NHDU while at the same time markedly, reducing socioeconomic burden on government and family members.

The data from the present study, showed the mean of injury severity score was 27.1 for ETG and 28.1 for LTG. Both ETG and LTG were indicated for tracheostomy based on their ISS, according to Gurkin et al. (2) which stated that; one of the indicators for tracheostomy was an ISS more than 25.0.

Questions might arise regarding the safety of a tracheostomy which can cause hypoventilation, hypercarbia, hypoxemia and arterial hypertension during the procedure especially when a patient is still on cerebral resuscitation. In defence of that, Stocchetti et al. (12) concluded that a translaryngeal tracheostomy is safe for the majority of the patients. However, the risk of intracranial pressure (ICP) must be taken into consideration and strict monitoring must include ICP, so that the benefits of early tracheostomy are not outweighed by the danger of intracranial decompensation.

## Conclusion

An early tracheostomy is indicated for patients with severe TBIs in order to reduce length of NHDU stays, the length of mechanical ventilator use and to reduce rate of VAP.

## Limitations and Future Recommendations

The study’s limitations were its small sample size and limited patient follow-up. In the future, the functional outcome of these patients according to the Glasgow Outcome Scale (GOS) needs to be analysed in order to determine the effectiveness and safety of this practice, i.e. patients need to be followed up for a longer time. Furthermore, a bigger sample size involving multiple centres is also needed to fully understand the impact of early tracheostomies on patients, socioeconomic factors and neurosurgery practices. As seen in Table 3, a large 95% CI: 1.92, 24.86 and 95% CI: 1.27, 17.41, respectively, for comorbidities and tracheostomies were due to reviewing only 19 cases of VAP (12.3%).

## Figures and Tables

**Table 1:** Characteristics of study population (*n* = 155)

Variables/Characteristics	Mean (SD)	*n* (%)
Gender		
Male		135 (87.1)
Female		26 (12.9)
Race		
Malay		95 (61.3)
Chinese		38 (24.5)
Indian		6 (3.9)
Others		16 (10.3)
Age	37.5 (16.7)	
GCS before tracheostomy (motor component)		
5–6		90 (58.1)
3–4		44 (28.4)
1–2		21 (13.5)
GCS after tracheostomy (motor component)		
5–6		110 (71)
3–4		32 (20.6)
1–2		13 (8.4)
Days in NHDU	7.44 (3.464)	
Duration on ventilator (days)	4.25 (2.508)	
Lung disease		
No lung disease		142 (91.6)
With lung disease		13 (8.4)
Pupil		
Equal		112 (72.3)
Unequal		43 (27.7)
Tracheostomy		
Early		72 (46.5)
Late		83 (53.5)
Injury severity score		
Early tracheostomy	27.07 (0.469)	
Late tracheostomy	28.13 (0.438)	
Marshal classification		
Type 2		3 (1.9)
Type 3		72 (49.7)
Type 4		11 (7.1)
Type 5		64 (41.3)
Pneumonia		
VAP		19 (12.3)
No VAP		136 (87.7)
Complication		
Infected		1 (0.6)
Subcutaneous emphysema		3 (1.9)
Status at discharge		
Alive		148 (95.5)
Dead		7 (4.5)

**Table 2:** Comparison between the outcomes early and late tracheostomy

Variables	Early tracheostomy mean (SD)	Late tracheostomy mean (SD)	Mean diff (95% CI)	*t* statistics^a^	*P*-value
Days in NHDU	4.75 (1.98)	9.77 (2.70)	−5.021 (−5.77, −4.28)	−13.313	0.004
Days on ventilator	2.65 (1.57)	5.63 (2.35)	−2.98 (−3.62, −2.33)	9.370	< 0.001

Note:

a Independent *t*-test was applied

**Table 3:** Association related variables towards presence of VAP

Variables	Simple logistic regression		Multiple logistic regression^c^	
	
	Crude OR (95% CI)	*P*-value	Adjusted OR (95% CI)	*P*-value
Age	1.02 (0.99, 1.05)	0.272		
Gender				
Male	1			
Female	0.00 (0.00, 0.00)	> 0.950		
Ethnic				
Malay	1			
Non-Malay	1.50 (0.57, 3.94)	0.410		
Comorbid				
No lung disease	1		1	
Lung disease	8.51 (2.49, 29.12)	0.001	6.92 (1.92, 24.86)	0.003
Smoking				
No	1			
Yes	1.04 (0.38, 2.80)	0.945		
Tracheostomy				
Early	1		1	
Late	5.49 (1.53, 19.72)	0.009	4.71 (1.27, 17.41)	0.020
GCS on admission				
5–6	1			
3–4	1.03 (0.36, 2.94)	> 0.950		
1–2	0.33 (0.04, 2.65)	0.294		
ISS	0.93 (0.81, 1.07)	0.328		
Pupil				
Equal	1			
Unequal	1.24 (0.44, 3.49)	0.690		

Note:

c Forward multiple logistic regression was applied
